# Comment on “Intratumorally specific microbial-derived lipopolysaccharide contributes to non-small cell lung cancer progression”

**DOI:** 10.1080/21505594.2026.2646024

**Published:** 2026-03-13

**Authors:** Eirini Anna Angelakopoulou, Ismini Maria Angelakopoulou, Fani Tsolaki

**Affiliations:** Faculty of Medicine, Aristotle University of Thessaloniki, Thessaloniki, Greece

Dear Editor,

We read with great interest the recent article by Sha et al. demonstrating that bacterial lipopolysaccharide (LPS) levels are elevated in non-small cell lung cancer (NSCLC) tumors when compared with adjacent non-tumor tissues, and that LPS can directly promote NSCLC cell proliferation both *in vitro* and *in vivo* by activating in tumor cells a TLR4-dependent signaling cascade involving mTOR, NF-κB and IL-6 [1]. These findings uncover a previously underappreciated role for LPS beyond classical infection contexts, with multifaceted implications for our understanding of microbiome – tumor relationships in NSCLC.

So far, numerous independent studies have reported enrichment with Proteobacteria (Pseudomonadota) in resected lung tumors, but the exact contributions of these microbes to lung cancer progression remain elusive [2]. By linking elevated intratumoral LPS levels to tumor cell proliferation through a well-characterized pro-inflammatory and growth-promoting signaling cascade, this work offers a plausible mechanistic explanation for these recurring observations. Importantly, Sha et al. move beyond descriptive associations to provide a novel, mechanism-driven framework for exploring tumor – microbiome interactions in lung cancer, in which microbial virulence factors such as LPS may be active players in tumor-promoting signaling, with potential implications for biomarker development and therapeutic targeting.

More broadly, this concept can be extended to other currently unexplored microbial factors in lung tumors. One interesting candidate in this microbiome context could be flagellin, the structural protein of flagella on several motile Proteobacteria frequently detected in human lungs, including lung tumors [2]. Flagellin is a known ligand for TLR5 on epithelial and immune cells, activating NF-κB and inducing pro-inflammatory cytokines (e.g. IL-6, IL-8) in a mechanism reminiscent of the LPS-induced cascade, while it can also recruit and modulate neutrophils and dendritic cells, further contributing to NF-κB – driven pro-inflammatory responses [3–6].

Future studies may build upon the pioneering work of Sha and colleagues by integrating bacterial isolation from tumor tissues, advanced culturomics and high-resolution metagenomics to characterize viable microbes inside tumors. Studying the structure and activity of tumor-derived microbial products will allow precise mapping of microbial immunostimulatory and virulence factors to specific oncogenic signaling responses in tumor or immune cells.

We congratulate the authors for this foundational contribution and hope this comment encourages further research into the complex interplay between microbiota, microbial virulence factors and signaling pathways in lung cancer.

Sincerely,

Eirini Anna Angelakopoulou, MSc

Ismini Maria Angelakopoulou, MSc

Fani Tsolaki, MD, PhD

## Data Availability

Data sharing is not applicable to this article as no new data were created or analyzed in this study.
